# Intimate partner violence against women and its association with pregnancy loss in Ethiopia: evidence from a national survey

**DOI:** 10.1186/s12905-020-01028-z

**Published:** 2020-09-04

**Authors:** Tenaw Yimer Tiruye, Catherine Chojenta, Melissa L. Harris, Elizabeth Holliday, Deborah Loxton

**Affiliations:** 1 Public Health Department, College of Health Sciences, Debre Markos University, Debre Markos, Ethiopia; 2 Research Centre for Generational Health and Ageing, School of Medicine and Public Health, Faculty of Health and Medicine, The University of Newcastle, Newcastle, Australia; 3 School of Medicine and Public Health, Faculty of Health and Medicine, University of Newcastle, Newcastle, Australia

**Keywords:** Intimate partner violence, Partner controlling behaviour, Pregnancy loss, Abortion, Stillbirth, Demographic and health survey, Ethiopia

## Abstract

**Background:**

Intimate partner violence (IPV) is major public health problem that affects many dimensions of women’s health. However, the role of IPV on women’s reproductive health in general and pregnancy loss in particular, is largely unknown in Ethiopia. Therefore, this study investigated the association between IPV and pregnancy loss in Ethiopia.

**Methods:**

A retrospective analysis of nationally representative data from the 2016 Ethiopian Demographic and Health Survey (EDHS) was conducted. Married women of reproductive age (15–49 years) who participated in the domestic violence sub-study of the survey were included in the analysis. Adjusted odds ratios were estimated using multilevel logistic regression models to represent the association of IPV with outcome variable.

**Results:**

Among 4167 women included in the analysis, pregnancy loss had been experienced by 467 (11.2%). In total, 1504 (36.1%) participants reported having ever experienced any form of IPV, with 25.1, 11.9, and 24.1% reporting physical, sexual and emotional IPV respectively. A total of 2371 (56.9%) women had also experienced at least one act of partner controlling behaviour. After adjusting for potential confounders, a significant association was observed between IPV (a composite measure of physical, sexual and emotional abuse) and pregnancy loss (Adjusted Odds Ratio (AOR) 1.54, 95% Confidence Interval (CI): 1.12, 2.14). The odds of pregnancy loss were also higher (AOR 1.72, 95% CI: 1.06, 2.79) among women who had experienced multiple acts of partner controlling behaviours, compared with women who had not experienced partner controlling behaviours. The intra-class correlation coefficient (ICC) indicated that pregnancy loss exhibits significant between-cluster variation (*p < 0.001*); about 25% of the variation in pregnancy loss was attributable to differences between clusters.

**Conclusion:**

IPV against women, including partner controlling behaviour, is significantly associated with pregnancy loss in Ethiopia. Therefore, there is a clear need to develop IPV prevention strategies and to incorporate IPV interventions into maternal health programs.

## Background

The aim of the current study is to examine pregnancy loss in relation to intimate partner violence (IPV) including partner controlling behaviours in Ethiopia. Pregnancy loss and IPV are both high in Ethiopia, but there is a lack of evidence regarding the relationship between the two major public health problems in the country. IPV is defined by the World Health Organization (WHO) as “any behaviour within an intimate relationship that causes physical, psychological or sexual harm to those in the relationship that includes acts of physical aggression, psychological abuse, sexual coercion and controlling behaviours” ([1] p89). For the purposes of this paper, pregnancy loss is defined as the termination of pregnancy either through abortion or stillbirth [2, 3]. Abortion is defined as the termination of pregnancy before foetal viability. Abortion can be either spontaneous (miscarriage), which is the termination of a pregnancy not provoked deliberately, or induced, which is voluntary termination of pregnancy [4]. Induced abortion is unsafe if it is performed in an unhygienic setting, by unqualified providers or without the necessary equipment [4]. Worldwide, the estimated annual number of unsafe abortions between 2010 and 2014 was 25.1 million, representing 45% of all abortions globally, and 97% of abortions in developing countries [5]. In Ethiopia, in 2014, it was estimated that 13% of all pregnancies ended with induced abortion [6]. Moreover, many women had unsafe abortions with about 47% of abortions not performed in a health facility [6]. Unsafe induced abortion is one of the five leading direct causes of maternal mortality in Ethiopia [7]. In addition to their effect on maternal mortality, abortions also contribute to other long-term adverse maternal health consequences, such as chronic pain, pelvic inflammatory diseases, ectopic pregnancies, infertility, and mental health problems [8].

Different socio-economic, demographic and fertility-related factors have been found to be associated with abortion. Factors commonly related to abortion are younger maternal age, unstable relationships, rape, family objection to the pregnancy, health concerns, non-use of contraception, contraceptive failure, lack of reproductive education, limited access to reproductive health services, and financial constraints [8–10]. IPV, which is also a global public health problem and affects one in every three women [11], has been identified as a factor associated with induced abortion [12, 13] and spontaneous abortion [14, 15]. However, there are some limitations in estimating the effect of IPV on abortion. For instance, it is more difficult for women to disclose induced abortion, especially if the abortion was illegal [16] or culturally unacceptable, [8] which might lead to bias. Additionally, there is no universal definition of abortion; for example, some countries define abortion as occurring before 20 weeks of gestation [15] or 22 weeks of gestation [17], while others use a 28 week threshold [14]. For this reason, women may also face difficulties identifying the technical distinction between miscarriage (involuntary termination of pregnancy before viability of foetus to survive out of the uterus) and stillbirth (involuntary termination after viability) [8, 18]. Due to this, some researchers have classified these outcomes as a composite measure using the term “pregnancy loss” [2, 3, 13, 19–21]. In studies conducted based on these composite measures, researchers have found a significant relationship between maternal IPV experience and pregnancy loss [2, 3, 13, 16, 19–22]. In communities with patriarchal norms, partner controlling behaviour may also affect women’s decision-making regarding fertility and use of contraception, which could also contribute to termination of pregnancy [22–24].

Ethiopia has one of the highest national rates of IPV, with the lifetime prevalence of IPV ranging from 20 to 78% in different areas of the country [25] and about 57% of women reporting having experienced at least one form of marital controlling behaviour from their partner [7]. There are no consolidated IPV prevention guidelines or services in the Ethiopian health system. Existing interventions are mainly for rape victims. Tolerant community norms with regard to IPV can lead individuals and organisations to disregard some acts of violence; norms of male superiority, and perceiving IPV as an inevitable part of a relationship allow IPV to persist in Ethiopian society and challenge intervention and prevention efforts [26]. In such contexts, a violent and/or controlling partner can further escalate the rate of abortion. In one small-scale study from west Ethiopia, significant relationship between women’s experience of IPV and termination of pregnancy (spontaneous/induced abortion) were identified [27]. However, there is no sufficient national level evidence regarding the role of IPV in pregnancy loss in Ethiopia.

The existing evidence regarding the effects of IPV on women’s health in Ethiopia is also limited. Existing studies have found associations between IPV and depression [28–31], psychiatric disorders [32], risk of acquiring sexually transmitted infections [27] and Human Immunodeficiency Virus (HIV) [33, 34], unmet need for family planning [35], unintended pregnancy [27], and low maternal health service utilization [36, 37]. Most of the studies have been conducted in small geographic locations that lack national level representativeness. Insufficient national level evidence might be one of the reasons that little attention has been given to IPV within maternal health programs. Therefore, the aim of this study was to investigate the association of IPV, including partner controlling behaviour, with pregnancy loss in Ethiopia.

## Methods

### Data source

This study used data from the 2016 Ethiopian Demographic and Health Survey (EDHS), which was the year the domestic violence module was added. The EDHS was a national survey conducted from 18 January to 27 June 2016. The 2016 EDHS data was collected with five questionnaires (household, women, men, biomarker and health facility).

### Sample size and sampling procedures

The EDHS used 84,915 enumeration areas; each enumeration area has an average of 181 households from nine regions and two city administrations. A two-stage stratified cluster sampling design was then implemented. First, 645 enumeration areas were selected from urban (202 enumeration areas) and rural (443 enumeration areas) areas based on proportional to size allocation. In the second stage, on average, 28 households per selected enumeration area were identified using systematic random sampling. All women aged 15–49 years in the household were eligible for the EDHS interview. Accordingly, 15,683 women, with a response rate of 95%, participated in the general survey [7].

For the domestic violence sub-study, only one married woman per household was interviewed. Of those women who were eligible, 97% (*n* = 5860) were interviewed, with 3% not involved mainly due to a lack of privacy. Background characteristics between selected women for the IPV sub-study and the general female population in the selected households was shown to be similar and did not reduce representativeness of the EDHS sample [7].

For this analysis, ever-married women who had complete data related to their pregnancy and birth history and responded to the IPV questionnaire were included. Women who had never been pregnant, who were missing either the outcome variable or IPV data were excluded from the analysis. Accordingly, 4167 (unweighted sample of 4372) women were included in the analysis.

### Measurement and variables

The outcome variable for this study was pregnancy loss. In the 2016 EDHS, women were asked a single question “Did you have any miscarriages, abortions or stillbirths that ended before 2011?” In addition, women were asked about their pregnancy and birth history during the 5 years (2011 to 2016) before the survey that provided information about whether the pregnancy was terminated or ended with a live birth [7]. Aggregating the responses from these two questions, women who had ever experienced pregnancy loss were identified. Accordingly, pregnancy loss was coded as ‘Yes’ if respondents reported ever having experienced a miscarriage, induced abortion, or stillbirth and ‘No’ if women had never experienced any of the three events. This method of defining pregnancy loss has been used in previous research [18, 20, 22].

The exposure variable was having ever experienced IPV (physical, emotional, and sexual violence, and partner controlling behaviour). IPV was measured based on women’s self-reported responses to questions asked whether or not they had experienced a number of violent acts within their relationship, perpetrated by their husband/partner for currently married women and recent husband/partner for previously married women (including widows). Physical IPV was assessed by asking participants seven questions regarding having: ever been pushed, shaken, or thrown something at her; slapped; her arm twisted or hair pulled; punched with fist or with something that could hurt; kicked, dragged, or beaten up; been choked or burnt on purpose; or been threatened or attacked with a knife, gun, or any other weapon. Three questions were asked to measure sexual IPV: having ever been physically forced to have sexual intercourse with her partner even when she did not want to, physically forced to perform any other sexual acts she did not want to, or forced with threats or in any other way to perform sexual acts she did not want to. Likewise, emotional IPV was assessed by asking three questions: if the participant had ever been humiliated, threatened, or insulted or made to feel bad about herself. Those women who were married more than once were also asked about spousal violence committed by any other husband/partner with two questions that asked about having ever been hit, slapped, kicked or done something else to hurt her and ever been physically forced to have intercourse or perform any other sexual acts against her will. Respondents were categorized as having experienced lifetime IPV if they had experience of any single act of physical, sexual or emotional IPV since the age of 15 years [7]. Likewise, any single act of partner controlling behaviour was categorized as ‘yes’ if one of the following behaviours were reportedly carried out on a woman by her husband: ‘being jealous if she talks to men’, ‘accusing her of being unfaithful’, ‘does not allow her to meet her friends’, ‘limits her contact with family’, and ‘tries to know where she is at all times’. Where women reported two or more acts of partner controlling behaviour, the responses were coded as ‘multiple controlling behaviours’ [7].

Variables that needed to be controlled in order to estimate the unbiased effect of the exposure upon the outcome were identified based on an examination of previous literature [2, 3, 8, 13, 16, 18–22, 27]. Accordingly, current age of the respondent (15–19/20–24/25–29/30–34/35–39/40–44/45–49 years), age at first cohabitation (< 15/15–18/≥18 years), respondent’s educational status (uneducated/primary/secondary+), religion (Christian/Muslim/other), number of children ever born (≤1/2–3/≥4) were considered. In addition, respondent’s employment status, rurality (urban/rural), region (11 administrative regions), decision-making, wealth index, media access, substance abuse, and pregnancy intention were included. Respondent’s employment status was grouped as employed/not employed based on their response to “have you been employed in the last 12 months”. Decision-making autonomy was coded as ‘yes’ if women reported being involved in all decisions regarding her own health care, major household purchases and visits to her family or relatives. Household wealth index was measured based on the number and kind of goods households have and housing characteristics (drinking water, toilet facility, flooring material and availability of electricity), and was generated using principal component analysis and classified into quintiles from 1 (very poor) to 5 (very rich). Media access was measured as whether the respondent read a newspaper, listened to the radio, or watched television and was categorized as no access, access less than once a week, and access at least once a week. Substance abuse was classified ‘yes’ if respondent drinks alcohol, chews khat (a green plant consumed as a stimulant) or smokes tobacco and ‘no’ otherwise. Pregnancy intention of respondents was categorized into two as ‘unintended’ and ‘intended’. A respondent was defined as having an unintended pregnancy if she had a pregnancy in the past 5 years that was either mistimed (wanted the pregnancy to happen later i.e. after 2 years) or unwanted (did not want the pregnancy at all).

### Data processing and analysis

Multilevel logistic regression models were fitted considering hierarchical nature of EDHS data (4167 women nested in 640 clusters). Multilevel analysis allows for the estimation of valid standard errors by adjusting for within-cluster correlation of the response variable [38]. Two models were constructed; Model I (the empty or unconditional model) and Model II (two independent models for IPV and partner control behaviours). In Model I, no independent variables were included. This model was used to estimate the random intercept at cluster level and the variation in pregnancy loss between clusters. Then, a second model was constructed by adding covariates and main independent variable (IPV or partner controlling behaviours) to Model I. Interactions between variables were assessed. Model fit was tested using Likelihood ratio test and the Akaike Information Criterion (AIC).

Model II was the final model used to estimate measures of association between IPV and pregnancy loss. Adjusted odds ratios together with the 95% CI were used to report associations. Statistical significance was declared using a *p*-value < 0.05. The measure of variance (random effects) was reported in terms of the intra-class correlation coefficient (ICC). The ICC measures the extent to which women within the same cluster are more similar to each other in the outcome variable (i.e. pregnancy loss) than they are to women in different clusters [38].

All the analyses took into account the EDHS sampling weight and were based on the weighted sample (*n* = 4167). The sampling weights used in the EDHS account for the complex sampling procedures (multi-stage stratified cluster sampling) that might cause an unequal probability of selection for certain areas or subgroups either due to design or coincidence. Hence, sampling weights were adjusted for differences in probability of selection and interview that allow extrapolation of results to the national level of representativeness [7].

## Results

### General characteristics of respondents

The majority of study participants were aged 25–29 years (22.4%), married before 18 years of age (63.6%), illiterate (63.6%), Christian (65.2%) or living in a rural area (82.7%). In total, 39.1% of participants reported having no decision-making autonomy, 47.9% of individuals described themselves as having a habit of substance abuse, and 63.1% had no access to media. In terms of regional context, about two third of participants were from Oromia (*n* = 1659; 39.8%) and Amhara (*n* = 966; 23.2%) regions whereas three regions (Dire Dawa, Gambela, and Harari) represents only 1% of study participants (Table 1).

**Table 1:** Participant characteristics (*n* = 4167)

Variable	Class	Weighted frequency	%
Current age	15–19	164	3.9
	20–24	573	13.8
	25–29	933	22.4
	30–34	885	21.2
	35–39	734	17.6
	40–44	498	11.9
	45–49	381	9.1
Age at first cohabitation	< 15 years	1111	26.7
	15–18 years	1539	36.9
	> = 18 years	1517	36.4
Educational status	No education	2650	63.6
	Primary	1080	25.9
	Secondary+	437	10.5
Employment status	Not employed	2023	48.5
	Employed	2144	51.5
Religion	Christian	2718	65.2
	Muslim	1375	33.0
	Other	75	1.8
Type of place of residence	Urban	719	17.3
	Rural	3448	82.7
Region of residence	Tigray	287	6.9
	Afar	38	0.9
	Amhara	966	23.2
	Oromia	1659	39.8
	Somali	122	2.9
	Benishangul	41	1.0
	SNNPR	885	21.2
	Gambela	12	0.3
	Harari	9	0.2
	Addis Ababa	127	3.1
	Dire Dawa	20	0.5
Decision-making autonomy	No	1630	39.1
	Yes	2537	60.9
Number of children ever born	One or less	777	18.6
	Two-three	1182	28.4
	Four or more	2209	53.0
Substance abuse	No	2171	52.1
	Yes	1996	47.9
Pregnancy intention (*n* = 2969)	Intended	2181	73.5
	Unintended	788	26.5
Wealth index	Poorest	806	19.3
	Poorer	799	19.2
	Middle	882	21.2
	Richer	789	18.9
	Richest	890	21.4
Access to media	No access	2631	63.1
	< once a week	611	14.7
	> = once a week	925	22.2

*SNNPR* Southern Nations, Nationals and Peoples Region

### Prevalence of different forms of IPV and pregnancy loss

Table 2 shows the estimated prevalence of different forms of IPV and pregnancy loss with 95% CIs. The least prevalent form of IPV was sexual IPV (*n* = 496, 11.9%), and the most prevalent form was partner controlling behaviour (*n* = 2371, 56.9%). About two in every three women had experienced at least one form of IPV in their lifetime. The number of participants who had experienced pregnancy loss was 467 (11.2%). Table 3 shows pregnancy loss by different participant characteristics.

**Table 2:** Prevalence of different forms of IPV and pregnancy loss in study participants (*n* = 4167)

Form of IPV	Weighted prevalence	95% CI
Physical IPV	25.1%	(22.8, 27.3%)
Sexual IPV	11.9%	(10.1, 13.7%)
Emotional IPV	24.1%	(21.7, 26.5%)
Physical, sexual or emotional IPV	36.1%	(33.4, 38.9%)
Partner controlling behaviour (single form)	56.9%	(54.1, 59.8%)
All forms of IPV	64.9%	(62.3, 67.5%)
Pregnancy loss	11.2%	(9.7, 12.7%)

*IPV* Intimate Partner Violence, *CI* Confidence Interval

**Table 3:** IPV experience by pregnancy loss (*n* = 4167)

Factor group	Variable	Class	Pregnancy loss		
			No	Yes	*P*-Value
			No (%)	No (%)	
Exposure variables	IPV	No	2401 (64.9)	260 (55.6)	0.009
		Yes	1299 (35.1)	207 (44.4)	
	Partner Controlling Behaviour	None	1629 (44.0)	166 (35.5)	0.081
		Single act	929 (25.1)	131 (28.1)	
		Multiple acts	1142 (30.9)	170 (36.4)	
Maternal characteristics	Current age	15–19	152 (4.1)	12 (2.6)	0.001
		20–24	526 (14.2)	47 (10.1)	
		25–29	862 (23.3)	71 (15.2)	
		30–34	779 (21.0)	106 (22.7)	
		35–39	645 (17.4)	89 (19.0)	
		40–44	419 (11.3)	78 (16.8)	
		45–49	317 (8.6)	64 (13.7)	
	Age at first cohabitation	< 15 years	963 (26.0)	148 (31.7)	0.184
		15–18 years	1381 (37.3)	158 (33.9)	
		> = 18 years	1356 (36.7)	161 (34.4)	
	Educational status	No education	2346 (63.4)	304 (65.1)	0.836
		Primary	961 (26.0)	119 (25.5)	
		Secondary+	393 (10.6)	44 (9.4)	
	Employment status	Not employed	1830 (49.4)	193 (41.4)	0.033
		Employed	1870 (50.6)	274 (58.6)	
	Religion	Christian	2391 (64.6)	327 (69.9)	0.248
		Muslim	1242 (33.6)	133 (28.4)	
		Other	67 (1.8)	8 (1.7)	
	Type of place of residence	Urban	630 (17.0)	89 (19.1)	0.465
		Rural	3070 (83.0)	378 (80.9)	
	Decision-making autonomy	No	1450 (39.2)	181 (38.7)	0.886
		Yes	2250 (60.8)	287 (61.3)	
Maternal characteristics	Region of residence	Tigray	241 (6.5)	46 (9.8)	0.004^¥^
		Afar	33 (0.9)	5 (1.1)	
		Amhara	833 (22.5)	133 (28.5)	
		Oromia	1517 (41.0)	143 (30.5)	
		Somali	109 (2.9)	14 (3.0)	
		Benishangul	39 (1.0)	2 (0.5)	
		SNNPR	790 (21.3)	95 (20.4)	
		Gambela	11 (0.3)	1 (0.2)	
		Harari	8 (0.2)	1 (0.2)	
		Addis Ababa	104 (2.8)	24 (5.1)	
		Dire Dawa	16 (0.4)	4 (0.7)	
	Number of children ever born	One or less	688 (18.6)	89 (19.1)	0.070
		Two-three	1079 (29.2)	102 (21.9)	
		Four or more	1933 (52.2)	276 (59.0)	
	Substance abuse	No	1938 (52.4)	233 (49.8)	0.477
		Yes	1762 (47.6)	234 (50.2)	
	Pregnancy intention*	Intended	2010 (74.3)	171 (64.7)	0.007
		Unintended	694 (25.7)	94 (35.3)	
Household characteristics	Wealth index	Poorest	702 (19.0)	104 (22.2)	0.430
		Poorer	705 (19.0)	94 (20.2)	
		Middle	792 (21.4)	91 (19.4)	
		Richer	719 (19.4)	70 (15.1)	
		Richest	782 (21.1)	108 (23.2)	
	Access to media	No access	2360 (63.8)	271 (57.9)	0.238
		< once a week	532 (14.4)	79 (16.9)	
		> = once a week	808 (21.8)	118 (25.2)	

*IPV* Intimate Partner Behaviour, *SNNPR* Southern Nations, Nationals and Peoples Region

^¥^*P*-value was based on Fisher’s exact test. *n = 2969

### The association of IPV with pregnancy loss

In the univariate analysis, a significant association was observed between pregnancy loss and any form of IPV (Crude Odds Ratio (COR) 1.48; 95% CI 1.10, 2.00) and multiple acts of partner controlling behaviour (COR 1.45; 95% CI 1.02, 2.05). However, there were no significant associations observed between women experiencing single act of partner controlling behaviour and pregnancy loss (COR 1.39; 95% CI 0.95, 2.02) (Table 4).

**Table 4:** Associations between IPV and pregnancy loss among reproductive age women (15–49 years) in Ethiopia from the multilevel model

Variable	Participant had experienced pregnancy loss			
	COR (95%CI)	*p*-value	AOR (95% CI)^a^	*p*-value
Experience of any form of IPV (physical, emotional or sexual)				
No	Reference			
Yes	1.48 (1.10, 2.00)	0.01	*1.54 (1.12, 2.14)*	*0.009*
Experience of partner controlling behaviour				
None	Reference			
Single act	1.39 (0.95, 2.02)	0.088	1.38 (0.85, 2.14)	0.198
Multiple acts	1.45 (1.02, 2.05)	0.037	*1.72 (1.06, 2.79)*	*0.028*
Random effects			Model I	Model II^a^
Community variance (SE) (IPV model)			1.23 (0.21)^*^	1.13 (0.65)^*^
ICC in community (%) (IPV model)			27.2	25.6
Community variance (SE) (Partner control model)			1.23 (0.21)^*^	1.08 (0.65)^*^
ICC in community (%) (Partner control model)			27.2	24.7
Test of Model fitness			Model I	Model II^a^
Likelihood ratio (IPV model)			− 1386.82	− 1324.30
AIC (IPV model)			2777.64	2724.58
Likelihood ratio (Partner control model)			−1386.82	−1324.84
AIC (Partner control model)			2777.64	2727.68

*IPV* Intimate Partner Violence, *COR* Crude Odds Ratio, *CI* Confidence Interval, *AOR* Adjusted Odds Ratio, *SE* Standard Error, *ICC* Intra-class Correlation Coefficient, *AIC* Akaike Information Criterion

^a^Adjusted for respondent’s age, age at first cohabitation, respondent’s educational status, employment, religion, place of residence, region, decision-making autonomy, number of children ever born, substance abuse, household wealth index, and media access

*P-value ≤0.05

The results of the multilevel logistic regression analyses are presented in Table 4. Model I shows that there was statistically significant variation in pregnancy loss between clusters (σ^2^ = 1.23, *p*-value < 0.001). The ICC showed that pregnancy loss within the same cluster had a higher clustering (ICC = 27.2%). After the inclusion of independent variables in Model II, the ICC indicated that about 25% of the variation in pregnancy loss was attributable to differences between clusters. In addition, after controlling for sociodemographic and fertility-related variables, significant associations were observed between experience of any form of IPV (AOR 1.54, 95% CI: 1.12, 2.14) and having experienced multiple acts of partner controlling behaviour (AOR 1.72, 95% CI: 1.06, 2.79) and pregnancy loss.

## Discussion

The current study is based on nationally representative Ethiopian data and revealed that IPV was associated with pregnancy loss (abortion, miscarriage, or stillbirth) after controlling for potentially confounding variables. Multiple, but not single, partner controlling behaviours were also associated with pregnancy loss. This is the first national level study to investigate pregnancy loss in relation to IPV in Ethiopia. The results make evident that maternal health programs in Ethiopia must design strategies to reduce IPV and extend the existed maternal health services to encompass IPV services.

The findings of this study revealed that experience of IPV was associated with pregnancy loss. This finding is in line with results from studies performed in Germany [22], Pakistan [2], Bangladesh [13, 16], Cameroon [3], Democratic Republic of Congo [19], Tanzania [20], Kenya [21], and west Ethiopia [27]. Previous studies have also shown that IPV was significantly associated with induced abortion [12, 13, 20] and miscarriage [14, 15]. It is important to note that there may be reverse causality between IPV and pregnancy loss, in that women who have experienced pregnancy termination might experience IPV [24]. For example, a longitudinal study from India indicated that a history of induced abortion increased subsequent sexual and verbal abuse [24]. This might especially occur in communities where women are considered responsible for poor reproductive health outcomes [20].

The current study has also demonstrated that the odds of pregnancy loss were higher among women who have experienced multiple acts of partner controlling behaviour. This finding is in line with results from a study conducted in India [23]. Other previous studies have also established that partner’s reproductive coercion against wives, such as forced sexual activity, forced contraception, and forced pregnancy, result in a higher likelihood of termination of pregnancy [39–41]. However, the way partner controlling behaviour was measured in these studies was different to the measures used in the current study. We measured partner control in a way that reflects the general autonomy of women in their relationship whereas previous studies measured ‘partner control’ using measures of women’s sexual and reproduction autonomy [39, 40]. Hence, the findings are not directly comparable.

In the current study, there was no significant relationship between a single partner controlling behaviour and pregnancy loss. The higher the number of partner controlling behaviours, the more severely a woman was being controlled; therefore, her autonomy in decision-making is lower, and her ability to control her fertility is more likely to be compromised compared with women not subjected to controlling behaviours. This can lead to unintended pregnancies that in turn may further increase the rate of induced abortion [6, 10].

**Fig. 1:**
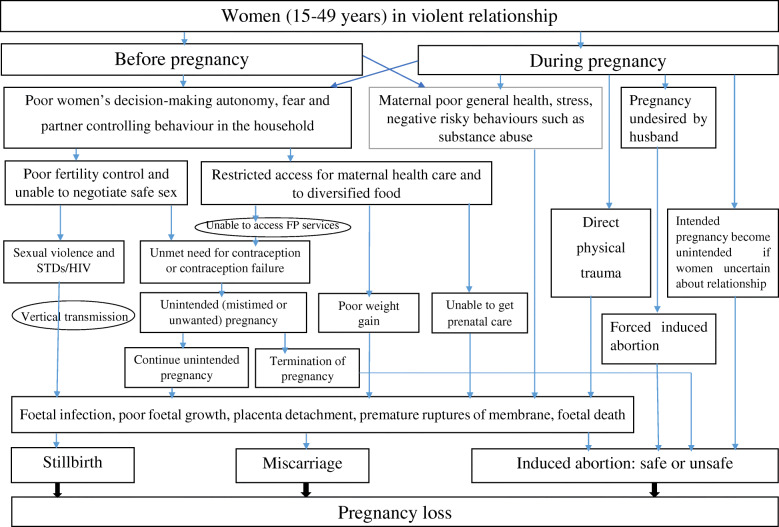
Potential pathways of the effect of maternal IPV victimization on pregnancy loss (adapted from 8, 14–16, 20, 22, 42–49)

Different potential direct and indirect pathways can be proposed for the observed associations between IPV and partner controlling behaviour with pregnancy loss. The direct pathways operate through injury, such as blunt abdominal trauma that leads to miscarriage [15, 16, 22] and through Sexually Transmitted Diseases such as HIV that occur due to sexual violence [42, 43] that in turn lead to foetal infection and miscarriage [14]. In addition, the mother’s poor mental or physical health that occurs due to IPV can cause poor foetal health [44, 45]. Withholding access to maternal health care due to partner controlling behaviour [20, 46, 47], or unwanted pregnancies that occur as a result of the inability to make decisions about fertility [8, 48, 49] can also explain the observed associations. The schematic representation of the possible pathways from IPV to pregnancy loss are presented in Fig. 1. While these have been demonstrated in past research, many of these pathways have not yet been examined in the Ethiopian context. Future longitudinal research is needed to verify and clarify the causal and temporal pathways between IPV and pregnancy loss. The current study has laid a foundation for this future work.

According to the UN report in 2016, Ethiopia’s Gender Inequality Index (GII) was 0.499 (ranked 116th of 188 countries), which is much higher than the perfect gender equality marker of GII of zero [50]. Therefore, as this study was conducted in a country where there is high gender inequality and patriarchal views [50], with high fertility desire among men more so than among women [7], men may threaten their wives and restrict them from accessing abortion services [51]. Due to this, women may not disclose abortion to their partner. When women attempt to hide access of abortion services from their abusive partner, they may be at increased risk of unsafe abortion [20]. Moreover, some women may not have been able to terminate their pregnancy safely due to beliefs that safe abortion is expensive or unavailable [10].

The Sustainable Development Goals with the theme of ‘no one should left behind’ [52] also put emphasis on improving gender equality. It is difficult to achieve this goal unless the issue of IPV is well addressed in the national programs of the country. Therefore, the outcomes of this study might be useful in developing comprehensive IPV prevention strategies and incorporate IPV interventions into existing maternal health programs. IPV interventions that focus on prevention, provision and protection (3Ps) can be put in place. With regard to prevention, the health sector should integrate IPV screening tools in maternal health care services and identify women who are experiencing IPV. The health sector can also play active roles in creating awareness about the consequences of IPV through school-based programs, community conversations and media. Other key interventions include counselling, medical care, and shelters. Case reporting systems and referral pathways are also needed to facilitate provision of these services. Intersectoral collaborations between justice, social and health systems can bring about systemic changes that protect women from IPV and promote women’s overall health. We also suggest policymakers, health care providers and stakeholders working in tackling IPV against women can use the evidence of the current study to underscore the need for funding such endeavours.

Lastly, this study revealed that variation in pregnancy loss was not completely explained by the included variables. This shows that some other community level variables and arcane social processes might be present. Therefore, future studies should focus on investigating community level factors and explore the social processes contributing to pregnancy loss. This is principally helpful for countries like Ethiopia, which contains a population of over 80 ethnic groups living in diverse contexts. These findings also suggest combined interventions directed at different community groups and regions may help to reduce pregnancy loss in this country.

### Strength and limitations of the study

The current study reveals the first Ethiopian national evidence that demonstrates the association between IPV and pregnancy loss, which is based on nationally representative and high quality data that were collected with well-trained data collectors, standardized measurement tools, and firm consideration of research ethics. This study has also provided new evidence that partner controlling behaviour, as a form of IPV, increases the odds of pregnancy loss. However, the study has limitations. First, the cross-sectional nature of study precludes the analysis of cause and effect relationships, as previously mentioned. Similarly, since both the exposure (IPV) and outcome (pregnancy loss) variables were measured retrospectively, it is possible that the outcome preceded the exposure in some cases. Second, although potential confounding variables were included, there could still be some residual confounding effects. For example, some factors occurring during pregnancy such as foetal conditions, maternal nutrition and weight gain, and infections were not assessed in this study due to these variables not being recorded in the dataset. The ICC estimates also suggest that the included variables did not completely explained the variation in pregnancy loss. Third, women may not want to report their experience of pregnancy loss due to distress or social reasons and under-reporting of IPV may occur due to fear of repercussions, stigma, or shame. However, the study has strictly followed WHO strategies for domestic violence research, which minimizes such under-reporting. Lastly, in Ethiopia, where patriarchal views are common, controlling behaviour is considered an acceptable behaviour for husbands in interactions with their wives [53, 54]. For this reason, women might not consider some controlling behaviours to be controlling, and this might led to underreporting.

## Conclusions

This study provides an important public health message that pregnancy loss is associated with IPV and partner controlling behaviour. The study also showed that significant cluster-level effects were found. The observed association makes evident that interventions are needed to reduce IPV in Ethiopia; this could help to minimize poor maternal health outcomes resulting from pregnancy loss.

## Data Availability

The dataset supporting the conclusions of this article is freely available to the public at www.measuredhs.com and can be accessed after request is made to and approved by the Demographic and Health Survey program.

## Abbreviations

AOR: Adjusted Odds Ratio
CI: Confidence Interval
COR: Crude Odds Ratio
EDHS: Ethiopian Demographic and Health Survey
HIV: Human Immunodeficiency Virus
IPV: Intimate Partner Violence
